# Dr. Whitlock Nicholl's General Elements of Pathology

**Published:** 1821-06-01

**Authors:** 


*General Elements of Pathology.* By WHITLOCK NICHOLL, M.D. M.R.I. A.F.I. of the Royal College of Physicians, London; Member of the Medical and Chirurgical Society; Corresponding Member of the Association of King's and Queen's College of Physicians, Dublin. Octavo, pp. 233. Callow, London, 1820.

DR. PARRY has justly observed in the preface to his Elements of Pathology and Therapeutics. "As the great and

---

ultimate end of pathology is its application to practice, it is by this criterion alone that the value of any pathological system ought to be estimated." The study of pathology has certainly gained more general attention from the junior branches of the profession, since the publication of Dr. Parry's Elements, and not before it was most seriously called for. Will it be credited, that only eight years ago, the views which are now generally entertained, and which dissection in a great measure has proved, of the relations existing between the vascular and nervous systems in health and disease, should have been ridiculed and laughed at? That in so short a period of time so great a revolution should have been effected in our pathological views, as to bring the majority of the *thinking* part of the profession to the same conclusions in regard to practice, is truly astonishing.—And in stating this, we have only to refer to the writings of the antients, and attend to the relations of dissections handed down to us, and wonder why, for so long a period, incorrect opinions should have been entertained. When perusing the works of Morgagni, we cannot but be suprized that the appearances upon dissection which are so faithfully and minutely detailed, should not have called forth some new light in the treatment of diseases, more particularly in those of a spasmodic nature. The bounden duty of every medical man is to think for himself, and to endeavour by indefatigable perseverance, in scrutinizing the wonderful mechanism of our frames, to arrive at just conclusions of what constitutes the difference between health and disease. Palmam qui meruit ferat. It is to Dr. James Sanders, we believe, that we are particularly indebted for many of the pathological opinions at present entertained. Dr. S. did not allow the observations of the antients to pass unnoticed, nor would he receive bare assertions for facts. During life, every symptom of disease was noted down, and after death, every part of the human body was carefully examined:—for instance, in convulsive diseases the muscles spasmodically affected were noted down; and after death, they, together with their nerves and blood-vessels, were carefully dissected, and compared with others in a sound state. These observations were more particularly made upon the human subject, both by himself and pupils. Dr. Moulson had an

---

the tarsus. As in the first case by removing a portion of the redundant skin we turn out the inverted tarsus, so in the latter by removing a portion of the elongated tarsus, we turn in the everted. It will be understood that it is only relatively that we speak of the elongation of the tarsus. It is everted, and strictured in the state of eversion by the skin."

opportunity of putting his views to the test, in five rabid dogs, independently of the numerous experiments he made upon horses and dogs in a healthy state. Dr. M. asserts that his practice and experiments are completely in unison. The observations on spasmodic and convulsive diseases by Dr. Moulson, which appeared in the third volume of the Monthly Series of this Journal, were the first which attracted particular notice, and novel as they then were supposed to be, was sufficiently proved by a contemporary editor demurring inserting them in his journal, because they were likely to induce a discussion: how far that may be we cannot say, but being well known to be Dr. Sanders's opinions, they were not liked.

Too much praise cannot be bestowed upon those individuals, who with their best endeavours, strive to add their mite to our circulating knowledge. It gives us pleasure to see men of talent, such as Dr. Nicholl, advancing just views of the human economy, and thereby establishing correct therapeutical knowledge. The plain and unostentatious manner in which these elements are ushered before the public, clearly realizes the motto affixed to the title page:—"Hoc opusculum ut in publicum ederem, non fecit profecto inanis, ac popularis auræ captandæ cupiditas, sed eo adductus sum, ut multis meorum æqualium hinc inde errantibus viam monstrarem, et aliquantulum munirem." This book is divided into sections, the first of which contains an outline of the human economy. "The organized body" is described "as consisting of a system of supply and waste; of a nervous system; of various assemblages of contractile fibres, which are called muscles; and of a fundamental structure, which consists of bones, of cartilages, and of the varieties of membrane." The system of supply and waste, consists of the vascular system, and of its two appendages, namely, the alimentary canal, and the pulmonary air-cavities. The various functions of the vascular and nervous systems, together with the respiratory and digestive organs, are here cursorily treated of, but under each distinct head, each division of the subject is regularly analyzed. These elements are written much after the manner of Dr. Parry's, but are not so fully treated of, as proofs and illustrations of what is advanced are not brought forward as in Dr. P.'s work. These are very necessary to the student, in order to command his attention, and give to him a taste of examining for himself. Simple ipse dixits do not pass so currently as in former times, and therefore it is necessary to bring conviction to the mind before any assertion can be received as true. In saying this, we by no means call in question the truth of Dr. Nicholl's views of the subject he treats of, for great praise is due to him for bringing together in so concise a form, a view of the functions of the human body.

According to Dr. N. no one part of the body is independent of another, and that it is by an equilibrium being sustained between the various functions of the human body that health is constituted. "For the functions of the vascular system would cease, if those of the nervous and muscular systems were suspended. The functions of the nervous systems would cease, if the offices of the vascular system were suspended. And the muscular system would be inert, were it not for the influence which it derives from the vascular and nervous systems." P. 14. In other words, in the chapter of general inferences, "there is a certain state of the several parts of the œconomy; a certain state of the several functions of each part; a certain mutual relation, and balance, and exchange of influence, between all these several parts and functions; which constitute that condition of living man to which the term *health* is applied." "The term disease embraces every state of the œconomy, which is different from that condition which is termed health."

The pathological views contained in this work are what are generally entertained by the profession at large. The manner in which they are drawn up alone differs—thus in describing congestion of the arteries of the brain: "should the tonicity of the cerebral arteries be so far lessened, that those vessels become over-distended with blood: or if, in consequence of the receipt of an increased quantity of blood by those vessels, an increased flow should take place from the cerebral exhalants; in either of these cases, inordinate compression of the cerebral substance may arise." P. 29. Arterial turgescence is but very rarely to be met with upon dissection; we in general only find the veins of the brain turgid. If an aneurism of the internal carotid arteries on the side of the sella turcica be alluded to, it is an exceedingly rare occurrence: the internal carotid arteries are very apt, in persons of an advanced age, to become ossified, and the same morbid change may be traced along their branches —in such cases, the deposit of ossific matter in the arteries would be a sufficient proviso against those vessels becoming over-distended with blood.

Dr. Nicholl seems to agree in opinion with those who believe that the generation of heat in the human body is dependent upon the combined action of the nervous and vascular systems. That it is so, there can be no doubt; yet, at the same time, we must not lose sight of the circulation on the brain and nerves. Two or three hemiplegic cases have occurred in our practice since Mr. Earle's cases and observations were published in the seventh volume of the Medico-Chirurgical Transactions, wherein the temperature of the paralized side considerably exceeded that of the unaffected side: the reverse generally being the case. How such was to be accounted for, it would be worse than useless to theorize about, as dissection, if any thing, could alone have afforded any clue to it.

"In many cases," Dr. N. says, "the act of retching is called forth by what I have termed revived sensation, as when the mention, or the sight of a substance, which has formerly excited vomiting, causes the act of retching to be reproduced, although there be no impression repeated on the stomach. Whatever produces great disgust, as the mention, or the sight, of a disagreeable substance which may never have been admitted into the stomach, may bring on retching." P. 109.

An illustration or two of the above would add a zest to the study of the subject, and engage more the attention of the junior members of the profession than the bare citation of the circumstance. We can mention one, out of many instances, where a medical friend, for some months after recovery from a fever, was thrown into a violent fit of retching at the mention of the word antimony: had he been in the midst of his dinner, or enjoying himself with his friends, an allusion to antimony would instantly induce retching. The manner in which muscular contraction is produced is thus stated:—

"The contraction of a muscle is produced by the transmission of nervous power to its fibres from cerebral substance, as from cranial, or spinal brain; this transmission is accomplished through the medium of nerves, along which we conceive the nervous power to be conveyed, in a direction from their cerebral to their anti-cerebral extremities. Whatever, then, causes the transmission of this power to muscles, will produce contractions of their fibres; and by whatever nerve this power is conveyed, contraction will take place in all those muscular fibres among which the anti-cerebral terminations of that nerve are distributed." P. 124.

We perfectly coincide with Dr. N. in his general pathological views; yet we cannot but repeat our regret at his not having advanced proofs and illustrations so as to have enlivened the subject. The dependence existing between the vascular and nervous systems may be proved in a variety of ways; and also, of muscular contraction being dependent upon the combined action of the vascular and nervous systems. Examples are continually occurring in the surgical department, in proof of what is advanced:—in the operation of popliteal aneurism, both the temperature and muscular action of the limbs are considerably altered by the division of the artery: the same follows the division of a nerve in any part of the body. That the nerves are dependent upon the vascular system for transmitting, what is termed, nervous power, may be proved by bleeding animals to death, as related by Drs. Moulson and Seeds.

Respecting the voluntary and involuntary muscles, the following opinion is entertained by some individuals:—that they are supplied by the same nerves, and that it is only by the stimulus applied to the involuntary muscles that keeps them in continual action. For instance, the heart is stimulated by the blood; withdraw that, and the heart ceases to act. The bowels act from the stimulus of their contents; the liver, &c. in the same way. The arteries in the arm and fore-arm running between muscles, are supplied by the same nerves as the muscles themselves. When the voluntary muscles are put into action, a greater quantity of blood is immediately directed to the nerves; and with the friction of the muscles, they give an increased influence to the muscles, so as to effect any object desired, in which force is required. The ganglia are not considered as so many small brains, but only binding the nerves in such a manner, that when an increased action is required, they act in the same manner as other nerves do when compressed by muscles:—for instance, when the intestines become filled with food, the nerves stretched upon them are drawn forwards, and these nerves, proceeding from ganglia, have their power increased in proportion as they are extended by the intestines, and bound down by ganglia. By disease, voluntary muscles become involuntary. In paralysis, apply galvanism to a leg or an arm, and immediately it acts. Subtract the stimulus of the blood from the arteries, and they become ligaments. So far it is considered as a proof, that involuntary actions in the different parts of the body are continued by the application of a stimulus, and that ganglia do not serve the office of minor brains, nor are there two sets of nerves in the human body, the one supplying the voluntary, and the other the involuntary muscles. How far the above conclusions may be correct, we do not take upon ourselves to determine; but shall leave so intricate a subject for those who may have more time than we have to theorize upon it.

In consequence of the intimate relation and connexion which subsist between all parts of the œconomy, Dr. N. considers it impossible to portray the various forms under which disease may appear, or to form any correct nosological system.

"There are two ways in which the bodily structure of man may be described. We may speak of it as being composed of distinct sets of structures, as of those which are comprised under the terms vascular system, and its appendages, nervous system, muscular system, and basal structure, which latter term comprehends the bones, the ligaments, and the varieties of membrane; or we may treat of it after the manner of geographers, as consisting of the head, of the neck, of the thorax, of the abdomen, and of the upper and lower extremities; subdividing each of these parts, and enumerating the several contents of each. An equal degree of mischief may result from each of these modes of considering our structure. For whichever mode we adopt, we acquire a habit of considering each portion which we enumerate as a distinct and insulated part. The consequence is, that when any deviation from the healthy state occurs in any one of the subdivisions which we have made, our attention is fixed upon the diseased condition of this particular portion of the body, while every other portion is supposed to preserve its former healthy state.

"Thus, those who have adopted the first mode of considering our bodily structure speak of diseases of the nervous system, diseases of the vascular system, and diseases of the muscular system. In so doing, they are incorrect, in as much as a disordered state of either of these systems implies also a diseased condition of each of the other systems.

"Those, on the other hand, who adopt the latter mode of regarding our bodily fabric, speak of diseases of the head, of the chest, of the abdomen, of the skin, &c. They subdivide these into diseases of the cranium, of the cranial brain, of the lungs, of the heart, of the liver, of the stomach of the bowels, of the spleen, of the pancreas, and so on. These persons act incorrectly also; because, in as much as, in each part which they enumerate, there are portions of the general nervous system, and of the general vascular system, it is evident that each part cannot be considered as a distinct insulated republic, but as a constituent portion of the general commonwealth, whose health is dependent upon a certain condition of every portion of the body." P. 143-4.

Were it possible to construct a correct nomenclature of diseased states of the human body, it would be truly desirable; but in our present state of knowledge, that is impossible. If the symptoms of disease were universally attended to, and not the name, more correct and fortunate practice would result therefrom.

Dr. N. perfectly coincides in opinion with those who will not allow dropsy to be a disease, but merely a consequence of various deviations from the healthy state. Indeed, ascites or anasarca, succeeding scarlatina, require very little medicinal treatment, provided the lancet has been used in the onset.

He strenuously admonishes the practitioner to guard against being governed in his practice by any nosological system, but seriously to attend to the symptoms as they present themselves, and to the junior members of the profession, in particular, we would recommend the following for their perusal.

"We must attend to every symptom, carefully investigating the causes of each, and from a general review of all the symptoms, and of all their several causes, we must endeavour to detect the primary source of the general disturbance, of which the symptoms are merely the sensible effects. The symptoms will be our guides in this research; and, as soon as we have discovered the object of our investigation, those guides should be dismissed from our notice. For in vain shall we combat effects, if the cause remain unsubdued." 153.

Annexed to these elements of pathology are three appendices; the first is an analysis of the phenomena of fever; the second are remarks on inflammation; and the third are aphorisms respecting absorption.

So much of late has been written both upon fever and inflammation, that we must refer our readers to Dr. Nicholl's work that they may duly appreciate it. After reviewing the several causes from which the symptoms of fever may arise, Dr. N. thus sums up:—

"It appears, then, that the several states which give rise to the several symptoms which accompany the progress of fever, may be arranged thus:—

| *In the Early Stage.* | *At the Height.* | *In the Decline.* |
|---|---|---|
| "Diminished sensibility of the nervous system. | Increased sensibility of the nervous system. | Diminished sensibility of the nervous system. |
| Diminished action of the heart. | Increased action of the heart. | Enfeebled action of the heart. |
| Contracted state of the small arteries. | Increased flow of blood through the smaller arteries; the opposition which is made by the exertion of the contractile power of these vessels, being overcome by the increased action of the heart. | Relaxed state of the small arteries." |

P. 177.

"The commencement and progress of fever are not always characterized by the train of states which have been laid down in the foregoing analysis. In some instances the symptoms which usher in fever, are such as denote the existence of increased sensibility of the nervous system, which may arise from an erethismal, or an inflamed, condition of the cerebral structures. The second stage of fever will, in these cases, be constituted by states similar to those which ushered in the attack; the difference being, that in the second stage they exist in a greater degree, and to a greater extent. The first and second stages of fever in these cases, then, differ in degree, not in essential character. The increased sensibility, in these cases, may proceed from erethism, to which inflammation may succeed, or the inflammatory state may exist from the commencement. The third stage, in these cases, may be characterized by states, similar to those which have been already pointed out as occurring in the decline of fever." 184.

The brief sketch which we have thus presented to our readers, we trust, will be a sufficient inducement for our brethren in the profession to have recourse to the work itself, in order that its merits, of which it possesses no small share, may be duly appreciated.

